# Intravascular guide wire aspiration in a patient on extracorporeal membrane oxygenation: A case report

**DOI:** 10.1097/MD.0000000000037638

**Published:** 2024-04-05

**Authors:** Jae Ho Choi, Jae Hwa Yoo, Won Ho Chang, Young Woo Park, Hyun Jo Kim, Hong Chul Oh

**Affiliations:** a Department of Thoracic and Cardiovascular surgery, Soonchunhyang University Hospital Seoul, Seoul, Republic of Korea; b Department of Anesthesiology and Pain Medicine, Soonchunhyang University Hospital Seoul, Seoul, Republic of Korea

**Keywords:** Case report, extracorporeal membrane oxygenation, guide wire

## Abstract

**Rationale:**

Guide wire aspiration during central venous catheter (CVC) insertion in a patient on extracorporeal membrane oxygenation (ECMO) is a very rare but dangerous complication. A guide wire aspirated inside the ECMO can cause thrombosis, the ECMO to break down or shut off, and unnecessary ECMO replacement.

**Patient concerns:**

A 58-year-old man was scheduled for venovenous ECMO for acute respiratory distress syndrome. After his vital signs stabilized, we inserted a CVC. During CVC insertion, the guide wire was aspirated into the ECMO venous line.

**Intervention:**

After confirming the guide wire inside the ECMO venous line, we replaced the entire ECMO circuit.

**Outcomes:**

ECMO was maintained for 57 days, and weaning was successful but the patient died 5 days afterward.

**Lessons:**

Care must be taken when inserting a CVC using a guide wire in ECMO patients: the guide wire should not be inserted deeply, it should be secured during insertion, the ECMO venous cannula tip requires proper positioning, and ECMO flow should be temporarily reduced.

## 1. Introduction

Patients with severe cardiopulmonary dysfunction sometimes require extracorporeal membrane oxygenation (ECMO). Most patients on ECMO require central venous catheter (CVC) placement for various purposes, such as fluid administration, vasopressor administration, or central venous pressure monitoring.^[1]^

Complications occur in about 15% of patients during CVC insertion; among these, loss of the entire intravenous guide wire is very rare.^[2,3]^ Although there are few reports of intravascular guide wire loss, it occurs more frequently in clinical practice than reported, possibly due to fear of legal issues.^[2,4]^ Although it is mostly asymptomatic, it can cause serious complications, including arrhythmias, vascular damage, and thrombosis. Additional evaluations to find the lost guide wire and additional procedures to remove it are also necessary.^[3,5]^

When this occurs in an ECMO patient, the guide wire can enter the ECMO circuit. This could cause the machine to suddenly shut down or the guide wire to remain inside the ECMO line, which could cause thrombosis. To remove the lost guide wire, the ECMO circuit must be replaced. To prevent this in ECMO patients, specific precautions are needed. To the best of our knowledge, however, there are only 2 case reports of guide wire aspiration in patients using ECMO.^[1,6]^ Therefore, we present a case of aspiration of the entire guide wire inside the ECMO circuit.

## 2. Case report

A 58-year-old man was found unconscious after vomiting in the bathroom and was admitted to the emergency room. He underwent neurosurgery for subarachnoid and intracerebral hemorrhages, and was being treated for accompanying aspiration pneumonia. However, his aspiration pneumonia worsened and he was treated with venovenous ECMO for acute respiratory distress syndrome after 1 month of hospitalization.

An ECMO venous line was inserted into his right femoral vein and an ECMO arterial line was placed in the right jugular vein; ECMO was maintained at 2970 rpm for a flow of 3.86 liters/minute. A few hours later, after his vital signs stabilized, a second-year resident in the cardiothoracic department attempted to place a CVC through the right subclavian vein. The resident had inserted more than 20 CVCs, including 5 in ECMO patients.

After venipuncture, a guide wire was inserted securely, and the dilator was used without any problems. After removing the dilator, the distal tip of the wire was held by hand and the catheter was pushed in. When catheter insertion was almost completed, the guide wire was suddenly sucked into the blood vessel, but there was no problem with CVC function.

An X-ray of the chest performed immediately did not reveal the lost wire. A few hours later, X-rays of the chest and abdomen were repeated and we checked the ECMO venous line. Initially, the wire was not visible in the ECMO line due to the dark venous blood, but when we bent the ECMO venous line slightly, the lost guide wire was page 5 overall confirmed inside the venous line.

**Figure 1.**
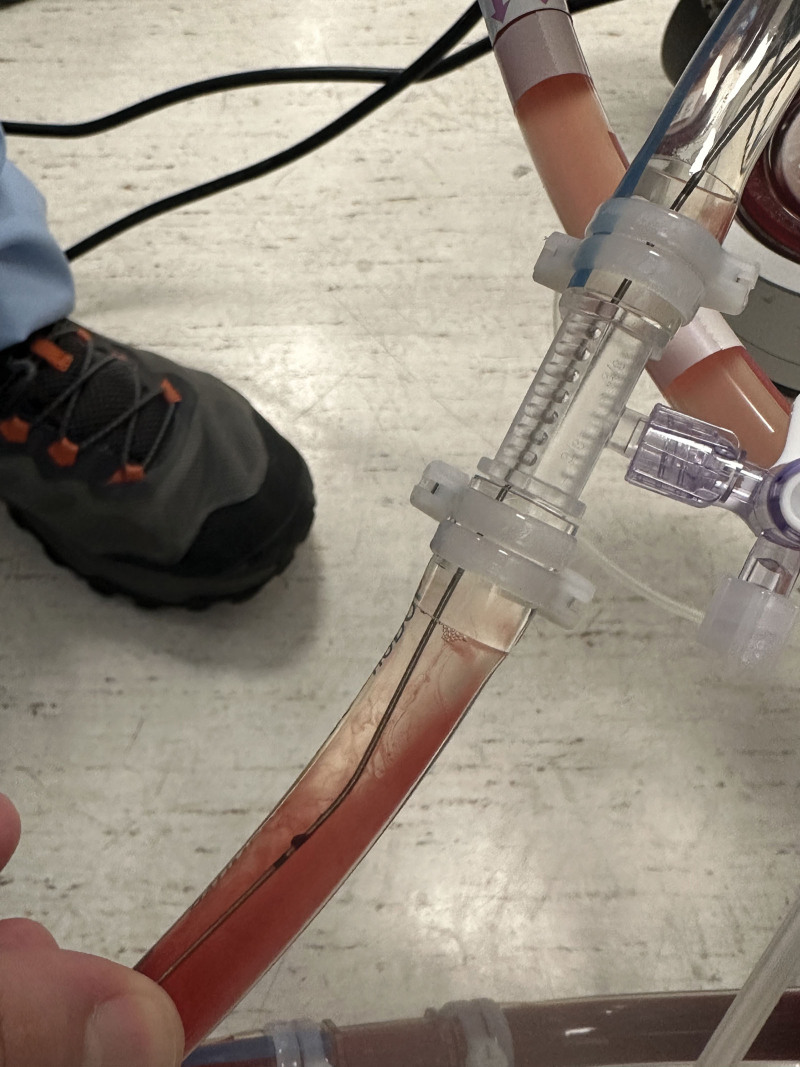
The lost guide wire in the venous ECMO line. Upon irrigating the removed ECMO circuit with saline, the lost guide wire was clearly visible.

We replaced the entire ECMO circuit and reconnected the venous and arterial cannulas. Upon irrigating the removed ECMO circuit with saline, the lost guide wire was clearly visible (Fig. 1),

We maintained this patient on ECMO for 57 days before successfully weaning him. Unfortunately, he died 5 days later.

Because the patient was deceased, informed consent could not be obtained from the patient. Therefore, a close relative provided informed consent for publication of the case. And this was approved by our hospital’s institutional ethics committee (SCHUH 2023-12-008).

## 3. Discussion

The main risk factors in typical cases of intravascular guide wire loss are unsupervised or improperly supervised CVC insertion by a trainee, distraction during CVC insertion, and a high workload.^[5]^ Up to 82.4% of these guide wires are lost by trainees who are new to the Seldinger technique.^[5]^ In ECMO patients, guide wire loss occurs because the wire is sucked in by the negative pressure of the ECMO venous cannula, rather than a simple mistake by an inexperienced trainee. Wire aspiration has even occurred in an ECMO patient when being performed by a senior consultant.^[1]^

To prevent intravascular guide wire aspiration, it is recommended that the guide wire not be pushed in more than 18 cm.^[7]^ In ECMO patients, however, loss cannot be prevented simply using this method. In our case, the guide wire was aspirated despite keeping it consistently shallower than 18 cm. Therefore, other factors also need to be considered.

Another recommendation to prevent guide wire aspiration is to hold on to its tip.^[3]^ However, this also appears to be insufficient for use in ECMO patients. In our case, guide wire aspiration occurred due to the negative pressure of the ECMO venous cannula at the moment when the proximal part of the guide wire could not be fixed during CVC insertion. To solve this problem, we will probably need to use 2 surgical clamps when inserting the catheter into the guide wire. First, 1 clamp is used to fix the proximal part, then the CVC is passed along the guide wire, and then the second clamp is used to hold the distal tip of the guide wire coming out of the CVC, while inserting the CVC.

The ECMO type or CVC puncture site does not appear to be a significant factor in guide wire aspiration. In the previous cases, the right jugular vein was punctured using venoarterial ECMO, whereas in our case the right subclavian vein was punctured using venovenous ECMO.^[1,6]^

Comparing the procedures during which time wire aspiration has occurred in previous reports, 1 previous case occurred immediately after guide wire insertion after venipuncture and the other occurred during use of a vein dilator.^[1,6]^ Our case occurred during the final stage of CVC insertion. Therefore, guide wire aspiration can occur at any time while the guide wire remains in the blood vessel for CVC insertion.

Presumably, when the end of the wire meets the entrance of the ECMO venous cannula, guide wire aspiration occurs instantaneously. Therefore, preventing contact between the guide wire tip and ECMO venous cannula orifice might be an important factor to prevent guide wire aspiration. In theory, placing the venous cannula in the middle of the right atrium instead of inserting it into the superior vena cava could reduce contact between the guide wire and venous cannula orifice. However, it has occurred even when it was located in the middle of the right atrium.^[6]^

As another method, temporarily lowering the ECMO flow during CVC insertion could be considered. During CVC insertion, the guide wire is inserted for less than a few minutes, so a temporary decrease in ECMO flow would not have a significant effect in a stable ECMO patient. In our patient, 4 more CVCs were inserted during the 57 days he was on ECMO, each time with the ECMO flow reduced and 100% oxygenation on the ventilator. The ECMO flow was reduced only during guide wire insertion after venipuncture, and this time did not exceed 5 minutes. As a result, no other complications occurred.

## 4. Conclusion

Wire aspiration during CVC insertion in ECMO patients is a very rare complication. However, when it occurs, it causes several problems with ECMO function and requires unnecessary replacement of the ECMO circuit. To prevent this complication, several precautions should be taken: the guide wire should not be inserted deeply, it should be secured during insertion, the ECMO venous cannula tip requires proper positioning, and ECMO flow should be temporarily reduced.

## Author contributions

**Writing—original draft:** Jae Ho Choi, Jae Hwa Yoo, Hong Chul Oh.

**Data curation:** Jae Hwa Yoo, Won Ho Chang, Young Woo Park, Hong Chul Oh.

**Formal analysis:** Jae Hwa Yoo, Hyun Jo Kim, Hong Chul Oh.

**Writing—review & editing:** Jae Hwa Yoo, Hong Chul Oh.

**Conceptualization:** Hong Chul Oh.

**Funding acquisition:** Hong Chul Oh.

**Visualization:** Hong Chul Oh.
